# Duration of the Flaxseed Supplementation Affects Antioxidant Defence Mechanisms and the Oxidative Stress of Fattening Pigs

**DOI:** 10.3390/vetsci10090586

**Published:** 2023-09-21

**Authors:** Anna Sobeková, Elena Piešová, Zuzana Maková, Renáta Szabóová, Drahomíra Sopková, Zuzana Andrejčáková, Radoslava Vlčková, Dominika Faixová, Zita Faixová

**Affiliations:** 1 Department of Chemistry, Biochemistry and Biophysics, University of Veterinary Medicine and Pharmacy in Košice, Komenského 73, 04181 Košice, Slovakia; anna.sobekova@uvlf.sk; 2 Department of Biology and Physiology, University of Veterinary Medicine and Pharmacy in Košice, Komenského 73, 04181 Košice, Slovakia; elena.piesova@uvlf.sk (E.P.); zuzana.makova@uvlf.sk (Z.M.); renata.szaboova@uvlf.sk (R.S.); drahomira.sopkova@uvlf.sk (D.S.); zuzana.andrejcakova@uvlf.sk (Z.A.); radoslava.vlckova@uvlf.sk (R.V.); 3 Department of Pharmaceutical Technology, Pharmacognosy and Botany, University of Veterinary Medicine and Pharmacy in Košice, Komenského 73, 04181 Košice, Slovakia; dominika.faixova@uvlf.sk

**Keywords:** antioxidant parameters, blood, flaxseed, pig, tissue

## Abstract

**Simple Summary:**

Polyunsaturated fatty acids, particularly omega 3, have beneficial effects on health and disease prevention. Flaxseed is the richest terrestrial source of omega-3 fatty acids, especially alpha-linolenic acid, and is available for animal and human consumption in various forms. This study aims to evaluate the effect of the duration of a flaxseed diet on the antioxidant defence mechanisms of fattening pigs. Blood and heart, muscle, liver and brain tissues antioxidant parameters were measured after a 3- and 6-week flaxseed diet. We found that the effect of a feeding diet containing flaxseed is significantly conditioned by the length of the flaxseed addition.

**Abstract:**

This study was conducted to investigate the effect of the duration of a flaxseed diet on fattening pigs’ antioxidant defence mechanism in blood and tissues. Eighteen 20-week-old Landrace breed fattening pigs (BW 76.61 ± 2.30 kg) were divided into three groups of six animals. The control group was fed a basal diet. The FS3 group was fed the basal diet supplemented with 10% flaxseed for 3 weeks. The FS6 group received the same basal diet with flaxseed for 6 weeks. The total antioxidant capacity of the blood, measured as the total antioxidant status (TAS), total plasma antioxidant capacity (FRAP), reactive oxygen metabolites (dROMs) and total antioxidant capacity (PAT), was not affected by the flaxseed diet. The superoxide dismutase (SOD), catalase (CAT), glutathione peroxidase (GPx) and glutathione reductase (GR) activities were significantly decreased in the FS3 pigs in the heart (*p* < 0.05). However, in the FS3 group, the glutathione-S-transferase (GST) activity significantly increased compared to the control, but in the FS6 group, the activity was inhibited (*p* < 0.05). In the muscle, the CAT and GST activity was significantly decreased in the FS3 group (*p* < 0.05). The thiobarbituric acid reactive substance (TBARS) content was significantly reduced in the brain, muscle and heart in the FS3 group(*p* < 0.05). In FS6, the TBARS content significantly increased in the heart and brain (*p* < 0.05). Our results showed that the health effect of a flaxseed diet is significantly conditioned by the length of the flaxseed addition.

## 1. Introduction

Flaxseed, one of the oldest plants grown for oil and fibre, is of great importance in nutrition and disease prevention due to some of its health benefits [1]. The benefits of consuming flaxseeds are associated with the presence of chemical compounds with specific biological activity and functional properties [2]. In addition to the substances associated with positive health effects, flaxseeds also contain substances with adverse effects on health [2]. In general, one whole flaxseed contains 35–45% fat, 20–35% dietary fibre, 20–30% protein, 4–8% moisture, 3–4% ash and 1% simple sugars [3,4].

Flaxseed is rich in lignans (up to 0.7–1.5% of the dry weight of the seed), which are responsible for its antioxidant activities [5]. The predominant lignan is secoisolariciresinol diglucoside [4,6]. It is the most studied flaxseed phenolic compound concerning its in vitro antioxidant potential. It exhibits antioxidant activity through either direct radical scavenging or through the inhibition of lipid peroxidation [7]. Secoisolariciresinol diglucoside is converted into mammalian lignans, enterodiol and enterolactone by colon bacteria [7]. The absorption of enterolignan precursors from flaxseed has been shown to increase when the seeds are crushed or ground [8]. Phenolic acids (8–10 g/kg) and flavonoids (35–70 mg/100 g) are two of the other phenolic compounds that are present in flaxseed [2,4,9].

Flaxseeds contain antinutrients that may adversely influence health [10]. Cyanogenic glycosides are the major antinutrients of flaxseed [4]. The other anti-nutritional substances present in flaxseed include phytic acid, which can reduce mineral bioavailability due to its strong chelating properties, linatine, which can act as a vitamin B6 antagonist and trypsin inhibitors. [10,11].

Flaxseeds contain 9–10% saturated fatty acids, about 20% monounsaturated fatty acids and a high proportion of polyunsaturated fatty acids (PUFAs) [1,4,12]. Flaxseed is the richest plant source of omega-3 PUFAs, especially alpha-linolenic acid (ALA) [13]. Moreover, the lipids in flaxseed provide an excellent omega-6:omega-3 fatty acid ratio of 0.3:1 [2]. The omega-3 and omega-6 polyunsaturated fatty acids are considered biological antagonists because they compete as substrates of the same enzyme systems [14]. High levels of omega-6 fatty acids in the diet have been suggested to inhibit the metabolism of omega-3 fatty acids and negatively affect health [15].

There is increasing evidence that the inclusion of flaxseed or flaxseed oil in the diet can produce significant beneficial health effects in animals and humans through its anti-inflammatory effect, anti-oxidative capacity, lipid profile modulating properties and anti-tumour activity [1,11,16,17].

In pigs, dietary flaxseeds or flaxseed oil has been used to improve the oxidative stability of pork [18], to treat constipation and diarrhoea [19] or to treat neonates with intrauterine growth retardation [20].

In the diet of boars, flaxseed oil (3%) has been shown to improve their semen quality parameters and fertility by improving the antioxidant capacity and altering the fatty acid composition of seminal plasma in a subtropical climate [21].

However, the high content of ALA makes flax highly susceptible to oxidation, and, therefore, feed enriched with large amounts of PUFAs can be prone to oxidation and may increase oxidation reactions, leading to oxidative stress [22].

A meta-analysis by Musazadeh et al. [23] showed that flaxseed supplementation for < 8 weeks significantly reduced the malondialdehyde (MDA) level, which is a marker of oxidative stress, whereas interventions for > 8 weeks did not. Moreover, Shahid et al. [24] reported that increasing the duration of a flaxseed diet (10%) with vitamin E for more than 10 days had a mild adverse effect on a duck’s performance. Based on the available data in the literature on the effects of dietary flaxseed on animals and humans, we hypothesised that the antioxidant status of pigs may change depending on the length of time they are fed a diet supplemented with flaxseed.

Therefore, the aim of the present study was to evaluate the effect of the long-term (3 or 6 weeks) supplementation of ground flaxseed (10%) to the fattening pig´s diet on the antioxidant status.

## 2. Materials and Methods

### 2.1. Animals, Diets and Management

The experiment was carried out under the European Directive on the Protection of Vertebrate Animals used for Experimental and Other Scientific Purposes (Directive 2010/63/EU; 2010).

Eighteen Landrace breed fattening pigs (gilts) housed at the Pig Fattening and Slaughter Station Inc. (Vajanského Street 789, Spišské Vlachy, Košice) were used in the experiment, which lasted six weeks. The pigs were 20 weeks old and had an average live weight of 76.61 ± 2.30 kg at the beginning of the experiment. Animals were kept in pens (2 animals per pen; 4.3 m^2^) equipped with nipple drinkers (the average temperature was 18 °C; the humidity was 60%). Pigs were divided into three groups. The control group (C) was fed the commercial complete feed mixture for fattening pigs (Dom krmív, Spišské Vlachy, Košice) at a dose of 3 kg/head/day. The second group (FS3) was fed the same commercial feed mixture with a 10% flaxseed addition (Libra variety, ALA content 57% in total fat) for 3 weeks. The third group (FS6) was fed the same complete mixture enriched with 10% flaxseed for 6 weeks. There were six animals in each experimental group. The chemical composition of the diets is presented in Table 1. Animals had free access to water. The average weight of the animals at the end of the experiment (the day of slaughter) was 118.16 ± 4.84 kg. The health status of animals and the consistency of faeces were monitored daily.

### 2.2. Sampling and Analysis of Blood Parameters

The blood samples were taken after 3 weeks (FS3), after 6 weeks (FS6), or before slaughter (C). Blood from the *anterior vena cava* was collected in plastic tubes with K_2_EDTA/heparin as an anticoagulant or without an anticoagulant, according to the needs of the assays. Blood samples were centrifuged at 1419× *g* and 4 °C for 20 min [25]. Blood serum and blood plasma were stored at −18 °C until analysed. Aliquots of heparinised blood were immediately frozen at −80 °C until analysis.

The total plasma antioxidant capacity (FRAP, μmol/L) was determined in K_2_EDTA plasma by the ferric-reducing ability of the plasma assay published by Benzie and Strain [26]. The total antioxidant status (TAS, mmol/L) in heparinised plasma [27] was analysed using commercial kit TAS (Randox, UK). The measurements of reactive oxygen metabolite (dROMs) levels and total antioxidant capacity (PAT) were conducted using commercial kits (Diacron, Italy), according to the manufacturer’s instructions. The principle of the dROMs test is to measure the concentration of hydroperoxides, which are present in the sample, using the Fenton reaction. The results are expressed in the UCARR unit (equivalent to 0.08 mg/dL of hydrogen peroxide). The PAT test is an automated test designed to assess the antioxidant capacity of plasma by measuring its ferric-reducing ability. The results of the PAT test are expressed in a unit equivalent to 1.4 μmol/L of ascorbic acid.

The activity of glutathione peroxidase (GPx) in heparinised blood was assessed through the kinetic method [28] using the RANSEL kit (Randox, UK).

### 2.3. Sampling and Analysis of Tissue Oxidative Parameters

After slaughter, the animal liver, brain, muscle and heart samples were collected, after 3 weeks (FS3) or after 6 weeks (FS6) and (C), washed with cooled phosphate-buffered saline and frozen at −50 °C. The frozen muscle, liver, brain and heart tissues were cut into pieces and homogenised in a 5 mmol·dm^−3^ Tris-HCl buffer pH 7.8 containing 0.15 mol·dm^−3^ KCl, 1 mmol·dm^−3^ Na_2_EDTA and 2 mmol·dm^−3^ reduced glutathione using an Ultra-Turrax homogenizer. Homogenates (25% *w*/*v*) were centrifuged at 105,000× *g* for 60 min. and supernatants were stored at −50 °C until used for further analysis.

The activity of superoxide dismutase (SOD) was determined by measuring the inhibition of cytochrome c reduction using a xanthine/xanthine oxidase O_2_^•–^ generating system at 550 nm (25 °C) [29]. One unit of SOD activity was defined as the amount of enzyme that causes a 50% inhibition of cytochrome c reduction under the assay conditions. The catalase (CAT) activity was assayed through the decrease in absorbance of hydrogen peroxide at 240 nm (30 °C) [30]. The GPx activity in tissues was measured by monitoring the oxidation of NADPH + H^+^ at 340 nm, as described by Flohé and Günzler [31], in a coupled assay with glutathione reductase. Cumene hydroperoxide was used as a substrate. Glutathione reductase (GR) was determined by recording the decrease in NADPH + H^+^ absorbance at 340 nm due to GSSG reduction [32]. Glutathione-S-transferase (GST) was measured via the procedure of Habig and Jakoby [33] at 30 °C using CDNB as a substrate. One unit of enzyme activity (GPx, GR and GST) was defined as the amount of enzyme that catalyses the formation of 1 µmol of product per minute under the assay conditions.

Specific enzyme activities were expressed in U/mg of protein. The protein concentration was measured through the method of Bradford [34], using bovine serum albumin as a standard.

The determination of the content of reduced glutathione (GSH) in tissues was based on the reaction of thiol-selective Ellman’s reagent (2,2-dinitro-5,5-dithiobenzoic acid) with free SH groups to form a coloured product, which was determined spectrophotometrically at 420 nm [35]. Free thiol groups were in the protein-depleted sample, mainly in the molecule of GSH.

The lipid peroxidation products measured as thiobarbituric acid reactive substances (TBARS) were determined according to Gutteridge [36].

### 2.4. Statistical Analyses

The data analysis was carried out via Graph Pad Prism 5.0 for Windows (GraphPad Software, San Diego, CA, USA). The results of each variable are expressed as the mean ± SD (*n* = 6) of three independent determinations. A one-way analysis of variance (ANOVA) was used to evaluate the statistical significance using Tukey’s multiple comparisons between the control and experimental groups, with a significance level set at *p* < 0.05, *p* < 0.01 and *p* < 0.001.

## 3. Results

### 3.1. Total Antioxidant Capacity of the Blood of the Pigs

The data concerning the effects of the flaxseed diet’s duration on the blood’s total antioxidant capacity are listed in Table 2. The results revealed that the total antioxidant capacity of the blood was not significantly affected by the duration of the flaxseed diet.

### 3.2. Antioxidant Parameters in the Tissues

The effect of the duration of the flaxseed diet on the antioxidant parameters in the liver, the brain, the heart and the muscle are presented in Table 3.

In the FS3 group, a statistically significant reduction in the SOD, CAT, GPX and GR activity in the heart compared to the control was observed. In the FS6 group, the activity of these enzymes began to increase compared to the FS3 group, but their values were significantly lower than in the control group. The activity of GST, a detoxification enzyme, changed differently. In the FS3 group, the GST activity in the heart significantly increased compared to the control. The prolongation of the flaxseed diet administration (FS6 group) caused an inhibition of the GST activity compared to both the control group and the FS3 group.

**Figure 1:**
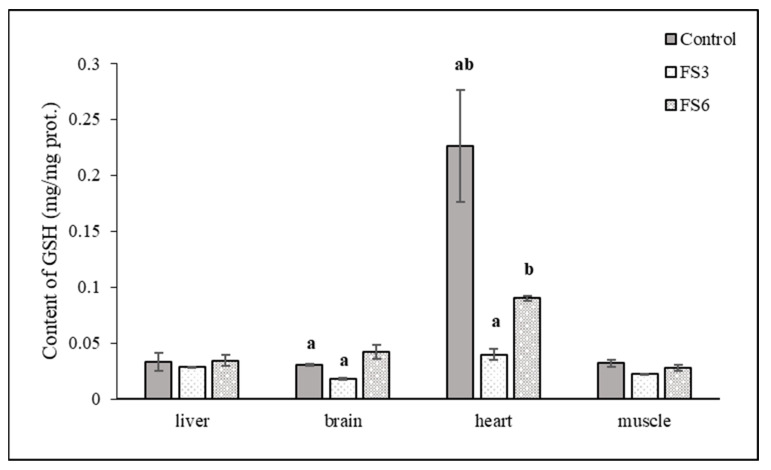
The effect of the duration of flaxseed supplementation in the diet on the level of glutathione in selected tissues of the pigs. Note: ^a,b^ means in the columns with the same superscripts are statistically significant (*p* < 0.05).

A three-week administration of the flax diet caused a statistically significant decrease in the specific activity of CAT and GST in the muscle. During another three weeks of the same diet, the activities of these enzymes rose to the level of the activities in the control group. The activities of the other enzymes were not significantly affected in the muscle during the experiment. Statistically significant differences in the activity of the antioxidant enzymes compared to the control group were not detected in the liver or brain either. GSH, as a co-substrate of GPx, GR and GST, plays an essential protective role against ROS. The changes in the level of reduced glutathione correlate with the observed changes in the activities of glutathione-dependent enzymes in the individual organs. A significant reduction in GSH level was observed in the heart in the FS3 group (Figure 1).

TBARS content, an indicator of the oxidative lipid damage, was reduced in the FS3 group for all the monitored organs. Except for in the liver, the reduction in TBARS content was statistically significant. The continuation of the diet containing flaxseeds caused an increase in TBARS content in all the organs (FS6 group). In the heart, the increase was statistically significant compared to the FS3 group, and in the case of the brain, the content of TBARS also significantly increased compared to the control.

### 3.3. Antioxidant Parameters in Tissues and Blood

As shown in Table 4, after three weeks of feeding the pigs a diet enriched with flaxseed, the activity of GPx in the blood was reduced. In the next weeks of feeding the same diet, the activity of GPx began to rise. The same tendency for changes was observed in the specific activity of GPx in organs. While the changes in the total GPx activity in the blood were not statistically significant, the changes in the specific GPx activity in the heart and muscle were significant.

## 4. Discussion

Due to its unique composition and resulting bioactivity, flax is used to enrich food to improve the nutritional quality of food. Flaxseed is commonly available for animal or human consumption in the form of whole flaxseed, ground flaxseed, flaxseed oil and a partially defatted flaxseed meal [11]. It is believed that the high content of polyunsaturated fatty acids in flaxseed is associated with an antioxidant system that is effective in preventing oxidation and maintaining the integrity of the seeds [37]. Antioxidant nutrients, such as vitamin E, protect cells from the damaging effects of free radicals. A study by Przybylski and Daun [38] indicated that tocopherols are probably not involved in the antioxidant system of flaxseed. One of the most common ways of evaluating the antioxidant effect of dietary supplements on the consumer is to monitor changes in the total antioxidant capacity of the consumer’s blood. In the experiment, we used four different tests of total antioxidant capacity. None of the tests used (TAS, FRAP, dROMs and PAT) confirmed that there were changes in the total antioxidant capacity of the blood of the experimental animals after they were fed with a diet containing flaxseed, regardless of the duration of the feeding (Table 2). Flaxseed contains cyclic hydrophobic linopeptides [11]. A peptide mixture with high levels of branched-chain amino acids and low levels of aromatic amino acids in flaxseed has shown antioxidant properties by scavenging DPPH [2]. The antioxidant activities of flaxseed gum, measured as DPPH, ABTS, reducing power, and total antioxidant activity, demonstrated the antioxidant potency of flaxseed gum [5]. In addition to polysaccharides, the oligosaccharides from flaxseed exhibit biological action. Liang et al. [39] reported a method of H_2_O_2_ oxidation to degrade flaxseed gum. The obtained flaxseed oligosaccharide exhibited a strong potential to scavenge hydroxyl radicals. Sembratowicz et al. [40] evaluated the effects of dietary flaxseed oil in an amount of 25 mL/100 kg BW/day on the antioxidant indices of horses. They reported that a 60-day diet containing flaxseed oil did not affect the total antioxidant potential (FRAP) of their blood. Króliczewska et al. [41] evaluated the effects of a diet with and without dietary flaxseed on the TAS levels in rabbits. There were statistically significant differences (*p* < 0.05) in the TAS parameter after 10 weeks of supplementation in the rabbits. The TAS in the blood plasma of rats receiving flaxseed cake was higher compared to rats fed the control diet without flaxseed supplementation. According to Singh et al. [21], a diet with 3% flaxseed oil did not affect the total antioxidant capacity (TAS) in boar serum.

Although the total antioxidant capacity in the blood did not change in our study, changes were observed in the antioxidant enzyme systems of selected organs of the experimental animals.

Reducing the TBARS content in all the organs in the FS3 group, along with the unchanged or reduced activity of antioxidant enzymes (Table 3), points to the protective antioxidant effect of flaxseed. Phenolic compounds are believed to be the main components responsible for the observed antioxidant activity of flaxseed [7]. The lignans in flaxseed have some antioxidant activity [5,42,43]. Through the further administration of a flaxseed diet, the activities of the monitored antioxidant enzymes in the individual organs were essentially adjusted to the level of activities set out in the control groups. The increase in the specific activities of antioxidant enzymes was associated with an increase in the content of TBARS, a marker of oxidative damage to lipids. These changes indicate that the protective antioxidant enzyme system of the organs responded to the presence of substances that could cause oxidative damage. The flaxseed phenols exerted either antioxidant or prooxidant activity. The strong antioxidant activity of the secoisolariciresinol in the nanoemulsions was attributed to its high free radical scavenging activity, but the flaxseed lignan exerted prooxidant activity in the nanoemulsions when it was completely hydrolysed and oxidised [44]. At present, there is no direct in vivo evidence regarding the effect of flaxseed phenols on raising the levels of endogenous antioxidant defences such as superoxide dismutase, catalase and glutathione peroxidase [7].

The liver is the primary organ involved in the metabolism and detoxification of xenobiotics. Hepatocytes and non-parenchymal liver cells activate the internal defence mechanisms of enzymatic and non-enzymatic detoxification aimed at neutralising damage in response to exposure to drugs, chemicals and xenobiotics. The determined specific activities of the antioxidant and detoxifying enzymes, as well as the TBARS values, confirmed this role of the liver. Our findings partially agreed with those of Shahid et al. [24], who reported that the breast muscle, liver and jejunal superoxide dismutase activity were not significantly affected by the duration (from 10 to 30 days) of a 10% flaxseed diet (*p* < 0.05), and malondialdehyde decreased linearly with the duration of the flaxseed diet in these tissues (*p* < 0.05) in Peking ducks. Naik et al. [45] investigated the effect of the supplementation of flaxseeds on hyperlipidemia in rats. The supplementation of flaxseeds reduced the level of TBARS in both the heart and liver tissues. This might be due to the antioxidant action of flaxseeds. Its antioxidant enzymes are capable of responding quickly in situations when cell damage is imminent [46]. The treatment of liver diseases with compounds of plant origin appears to be safe because they are “natural” and fit the image of a gentle and therefore harmless alternative to conventional medicine [47].

The brain is an oxygen-demanding organ. It uses up to 20% of the total oxygen consumption in the body. The brain has the second highest lipid content behind adipose tissue in an organism [48]. The differentiation and functioning of cultured brain cells require polyunsaturated fatty acids [49]. The ratio of omega-3 and omega-6 in the diet plays an important role in the enrichment of tissues. ALA is converted after its absorption into long-chain polyunsaturated fatty acids, such as EPA (20:5), omega-3 and DHA (22:6) and omega-3. An inadequate intake of omega-3 fatty acids decreases the amount of DHA and increases the amount of omega-6 fatty acids in the brain. Recent results have shown that a lack of ALA in the diet induces more pronounced abnormalities in cerebral structures than in other organs. Eckert et al. [50] found an increased amount of DHA in brain tissue phospholipids after the administration of ALA-rich flaxseed oil to rats. An increase in specific enzyme activities to the level of the control group was observed in the brain in the FS6 group. The activity of CAT and GST was higher than in the control group (CAT: 11.8→14.4 U·mg prot.^−1^; GST: 0.19→0.22 U·mg prot.^−1^). However, this increase was not significant. The higher activity of such enzymes during the experiment might also result from the body adapting itself to moderate regular oxidative stress conditions [51]. The decreased expression and decreased activity of SOD and CAT were observed in the brain of mice that were fed a long-term high-fat diet [52]. The TBARS content increased significantly not only compared to the FS3 group but also compared to the control group. This increase in the TBARS content indicates that, despite the increased activity of the mentioned enzymes (CAT and GST), oxidative damage to this organ occurred. In general, the polyunsaturated acids in lipids are easily oxidised to form hydroperoxides. These subsequently decompose into several secondary oxidation products. Lipid peroxidation in vivo can damage multiple organs in the body oxidatively. The results suggest that SOD, CAT and GST are involved in the regulation of oxidative stress in high-fat diet-induced brain damage.

Some chemical constituents of regularly consumed plants increase the activity or induce the expression of GSTs, a major family of enzymes involved in the biotransformation of both endogenous and exogenous toxic substances [53]. Thus, GST activity has been widely used as a biomarker for detecting oxidative stress in a wide range of organisms, including mammals and invertebrates [54]. Only in the heart tissue did we determine a statistically significant increase in the specific activity of this detoxification enzyme after 3 weeks of flaxseed diet administration. The increased GST activity was accompanied by significantly reduced GR activity, which ensures a sufficient level of reduced glutathione in the cell. The reduced GR activity in the heart was manifested by a significantly lower GSH level compared to the control group. GSH is one of the important antioxidants oxidised by ROS to GSSG, while GR, in turn, catalyses the GSSG to produce GSH [54]. The redox status of glutathione is highly essential for various biological events, and its homeostasis is critically regulated by GST activity. Any alteration in GSH metabolism and GST activity can affect various signalling events, leading to the development of different diseases [55]. The activities of the antioxidant enzymes were significantly reduced in the heart in the FS3 group. The lower SOD levels observed postnatally in goats were related to a lower production of reactive oxygen species in the body [56]. The reduced TBARS content in the FS3 group, despite the changes in the enzyme activities, indicates that the heart muscle was able to resist oxidative stress. GST activity was also inhibited after another three weeks of the same diet. Although the TBARS content in the FS6 group increased, it did not exceed the level of the control group. However, based on the observed trend in the changes in the monitored parameters, we can assume that a longer-term administration of such a diet would cause oxidative damage to the heart.

In the skeletal muscle of the experimental animals, no significant changes were recorded in any monitored parameter of oxidative stress (Table 3, Figure 1). However, the feeding of flax meal decreased the oxidative stability of broiler meat, reduced the free radical scavenging capacity of both fresh and stored meat and increased lipid oxidation in broiler meat during storage. The resulting oxidation products of the omega-3 and omega-6 fatty acids cause negative changes in the functional properties of myofibrillar proteins in processed muscle foods [57].

The GPx activity was determined not only in selected organs but also in the blood of the experimental animals. Glutathione peroxidase, as well as superoxide dismutase and catalase, are enzymatic antioxidants. GPx is a cytosolic enzyme and is responsible for the reduction in the amount of hydroperoxides in cells [58]. The results in Table 4 show that, just like the specific activity of GPx in the individual organs, the enzyme activity of GPx in the blood also changed. It was reduced in the FS3 group compared to the control and increased in the FS6 group. Unlike in the cardiac and skeletal muscle, these changes were not statistically significant.

Organisms have evolved specific mechanisms to protect themselves against oxidative stress, including non-enzymatic and enzymatic defences such as reduced glutathione and GSH-related enzymes. An efficient antioxidant system can, under normal physiological conditions, adjust the levels of oxidants in the body, metabolize excess free radicals, for example through CAT and SOD, and avoid potential damage [52]. In some pathological conditions of the organism, as well as in the case of increased intake of various biologically active substances, some aspects of the antioxidant system change. The latter cannot fully metabolize the active substances produced by the body, which reduces its ability to trap free radicals. An imbalance between the oxidant and antioxidant systems leads to oxidative damage to cells and has a fundamental role in the pathogenesis of many disorders.

## 5. Conclusions

Our results showed that feeding them a diet containing 10% ground flaxseed affected the antioxidant defence system of the experimental animals. The response of the antioxidant system was significantly influenced by the duration of the food supplementation.

Further studies in this field are needed to advance the recent knowledge of the biological actions of dietary flaxseed.

## Figures and Tables

**Table 1:** The nutritional composition of the standard feed mixture, flaxseed and combination (calculated to 100% of dry matter).

Variable	SFM	Flaxseed	SFM + 10% FS
Crude protein, g/kg	140.42	227.6	150.51
Crude fat, g/kg	18.07	307.25	42.23
Crude fibre, g/kg	35.69	232.58	68.29
NDF, g/kg	176.11	411.9	189.04
ADF, g/kg	42.27	278.9	74.47
Ash, g/kg	60.12	35.5	48.30
Starch, g/kg	574.62	42.32	510.28
Ca, g/kg	8.12	2.81	12.80
Mg, g/kg	3.35	4.11	3.59
Na, g/kg	1.45	4.33	1.52
K, g/kg	5.58	8.12	6.29
P, g/kg	4.80	2.71	5.84
Cu, mg/kg	40.82	33.55	57.40
Zn, mg/kg	131.83	48.7	103.45
Mn, mg/kg	150.57	43.83	125.80
ME, MJ/kg	13.26	12.87	13.36

Note: SFM, standard feed mixture; FS, flaxseed; NDF, neutral detergent fibre; ADF, acid detergent fibre; ME, metabolizable energy.

**Table 2:** The effect of the duration of flaxseed supplementation in the diet on the total antioxidant capacity of the blood of the pigs.

Item	Unit	Control	FS3	FS6
TAS	mmol/L	1.26 ± 0.08	1.12 ± 0.04	1.15 ± 0.04
FRAP	μmol/L	217 ± 16	212 ± 14	256 ± 33
dROMs	UCARR	1289 ± 70	1123 ± 31	1134 ± 65
PAT	1.4 μmol/L ascorbic acid	3196 ± 168	3288 ± 66	3368 ± 66

Note: Control, control group; FS3, experimental group, 3 weeks feeding a diet with flaxseed supplementation; FS6, experimental group, 6 weeks feeding a diet with flaxseed supplementation; TAS, total antioxidant status; FRAP, the total plasma antioxidant capacity; dROMs, reactive oxygen metabolites level; PAT, total antioxidant capacity. Results are expressed as mean ± SD. Means in a row with the same superscripts are statistically significant (*p* < 0.05).

**Table 3:** The effect of the duration of flaxseed supplementation in the diet on antioxidant parameters in the tissues of the pigs.

Item	Unit	Control	FS3	FS6
Liver				
SOD	U·mg prot.^−1^	102 ±12	81± 5	95 ±17
CAT	U·mg prot.^−1^	3650 ± 359	3930 ± 116	3820 ± 319
GPx	U·mg prot.^−1^	0.073 ± 0.017	0.047 ± 0.008	0.042 ± 0.007
GST	U·mg prot.^−1^	2.358 ± 0.237	1.451 ± 0.099	2.018 ± 0.223
GR	U·mg prot.^−1^	0.140 ± 0.022	0.121 ± 0.007	0.091± 0.013
TBARS	A_535_/mg prot.	0.0079 ± 0.0009	0.0057 ± 0.0003	0.0070 ± 0.0010
Brain				
SOD	U·mg prot.^−1^	43 ± 4	34 ± 2	39 ± 2
CAT	U·mg prot.^−1^	11.8 ± 0.2	10.8 ± 0.2	14.4 ± 0.2
GPx	U·mg prot.^−1^	0.015 ± 0.002	0.008 ± 0.002	0.014 ± 0.002
GST	U·mg prot.^−1^	0.190 ± 0.009	0.154 ± 0.012	0.220 ± 0.007
GR	U·mg prot.^−1^	0.0166 ±0.0012	0.0132 ±0.0006	0.0164 ± 0.0010
TBARS	A_535_/mg prot.	0.0078 ± 0.0002 ^a^	0.0019 ± 0.000 ^ab^	0.0098 ± 0.0007 ^b^
Heart				
SOD	U·mg prot.^−1^	231 ± 43 ^ab^	46 ± 2 ^ac^	75± 8 ^bc^
CAT	U·mg prot.^−1^	259 ± 41 ^ab^	79 ± 11 ^bc^	119 ± 7 ^ac^
GPx	U·mg prot.^−1^	0.178 ± 0.042 ^ab^	0.032 ± 0.001 ^a^	0.063 ± 0.005 ^b^
GST	U·mg prot.^−1^	0.216 ± 0.050 ^a^	0.369 ± 0.001 ^b^	0.077 ± 0.006 ^ab^
GR	U·mg prot.^−1^	0.177 ± 0.038 ^ab^	0.038 ± 0.006 ^ac^	0.059 ± 0.003 ^bc^
TBARS	A_535_/mg prot.	0.038 ± 0.013 ^ab^	0.005 ± 0.001 ^bc^	0.0103 ± 0.0024 ^ac^
Muscle				
SOD	U·mg prot.^−1^	9.2 ± 0.2 ^a^	8.6 ± 0.5	11.1 ± 0.3 ^a^
CAT	U·mg prot.^−1^	13.7 ± 0.6 ^a^	10.4 ± 0.7 ^a^	13.9 ± 2.3
GPx	U·mg prot.^−1^	0.009 ± 0.001 ^a^	0.009 ± 0.001 ^b^	0.015 ± 0.001 ^ab^
GST	U·mg prot.^−1^	0.032 ± 0.001 ^a^	0.024 ±0.001 ^a^	0.032 ± 0.004 ^b^
GR	U·mg prot.^−1^	0.0061± 0.0003	0.0060 ± 0.0004	0.0089 ± 0.0008
TBARS	A_535_/mg prot.	0.0076 ± 0.0008 ^a^	0.0050 ± 0.0006 ^b^	0.0068 ± 0.0004 ^ab^

Note: Control, control group; FS3, experimental group, 3 weeks feeding a diet with flaxseed supplementation; FS6, experimental group, 6 weeks feeding a diet with flaxseed supplementation; GPx, glutathione peroxidase; GST, glutathione-S-transferase; SOD, superoxide dismutase; GR, glutathione reductase; CAT, catalase; TBARS, thiobarbituric acid reactive substances. Results are expressed as mean ± SD. ^a,b,c^ means in a row with the same superscripts are statistically significant (*p* < 0.05).

**Table 4:** The effect of the duration of flaxseed supplementation in the diet on the activity of glutathione peroxidase in the blood and selected tissues of the pigs.

Item	Unit	Control	FS3	FS6
Liver	U·mg prot.^−1^	0.073 ± 0.017	0.047 ± 0.008	0.042 ± 0.007
Brain	U·mg prot.^−1^	0.015 ± 0.002	0.008 ± 0.002	0.014± 0.002
Heart	U·mg prot.^−1^	0.178 ±0.042 ^ab^	0.032 ± 0.001 ^a^	0.063 ± 0.005 ^b^
Muscle	U·mg prot.^−1^	0.009 ± 0.001 ^a^	0.009 ±0.001 ^b^	0.015 ± 0.001 ^ab^
Blood	μkat·L^−1^	715 ± 94 ^a^	386 ± 41 ^a^	575 ± 77

Note: Control, control group; FS3, experimental group, 3 weeks feeding a diet with flaxseed supplementation; FS6, experimental group, 6 weeks feeding a diet with flaxseed supplementation; GPx, glutathione peroxidase. Results are expressed as mean ± SD. ^a,b^ means in a row with the same superscripts are statistically significant (*p* < 0.05).

## Data Availability

All the data appear in the manuscript. For further inquiries, please contact the corresponding author.
